# Role of the Pharmacist in Managing Treatment-Resistant Depression: A Focus on Ketamine

**DOI:** 10.3390/pharmacy9030118

**Published:** 2021-06-25

**Authors:** Linda Xing Yu Liu, Marina Golts, Virginia Fernandes

**Affiliations:** 1 Department of Pharmacy, Mount Sinai Hospital, University of Toronto, Toronto, ON M5G 1X5, Canada; Virginia.Fernandes@sinaihealth.ca; 2 Leslie Dan Faculty of Pharmacy, University of Toronto, Toronto, ON M5S 3M2, Canada; 3 Department of Psychiatry, Mount Sinai Hospital, University of Toronto, Toronto, ON M5G 1X5, Canada; Marina.Golts@sinaihealth.ca; 4 Faculty of Medicine, University of Toronto, Toronto, ON M5S 1A8, Canada

**Keywords:** ketamine, esketamine, pharmacist, pharmacy practice, mental health, depression, treatment-resistant depression

## Abstract

The impact of depression is well described in the literature, and it is most prominent in patients who have trialed multiple treatments. Treatment-resistant depression (TRD) is particularly debilitating, and it is associated with significant morbidity and mortality. Despite this, there seems to be therapeutic inertia in adopting novel therapies in current practice. Ketamine is an N-methyl-D-aspartate receptor antagonist and anesthetic agent which has recently been shown to be effective in the management of TRD when administered intravenously or intranasally. The treatments, however, are not easily accessible due to restrictions in prescribing and dispensing, high costs, and the slow uptake of evidence-based practice involving ketamine within the Canadian healthcare system. Given the limited treatment options for TRD, novel approaches should be considered and adopted into practice, and facilitated by a multi-disciplinary approach. Pharmacists play a critical role in ensuring access to quality care. This includes dissemination of evidence supporting pharmacological treatments and facilitating translation into current practice. Pharmacists are uniquely positioned to collaborate with prescribers and assess novel treatment options, such as ketamine, address modifiable barriers to treatment, and triage access to medications during transitions of care. Extending the reach of these novel psychiatric treatments in both tertiary and primary care settings creates an emerging role for pharmacists in the collaborative effort to better manage treatment-resistant depression.

## 1. Introduction

Depression has a substantial impact on the emotional and functional well-being of those living with the condition, as well as their loved ones [1]. The impact of depression is well described in the literature, and it is most prominent in patients who have gone through multiple treatments without success [1]. Treatment-resistant depression (TRD) is particularly debilitating, and is defined in the American Psychiatric Association Diagnostic and Statistical Manual of Mental Disorders, Fifth Edition (DSM5), as inadequate response to two or more antidepressant trials of adequate doses and duration [2]. Approximately 10 to 30% of patients with major depressive disorder are described as treatment-resistant or treatment-refractory [3]. Among Canadian patients with depression, a study found that 22% of these patients in primary care settings were considered treatment-resistant [4]. Research has been conducted to attempt to quantify the overall burden of depression on the healthcare system. On an individual level, depression weighs heavily on one’s quality of life, impacting overall feelings of wellness and satisfaction. On a systems level, it has been found that much of the social and economic burden of major depressive disorder can be attributed to TRD, which is associated with a 50% increase in direct and indirect healthcare costs compared to non-resistant major depressive disorder [5,6,7].

Unfortunately, there are limited treatment options for patients with TRD. Current options include alternative or combination pharmacotherapy, and/or electroconvulsive therapy (ECT). A proportion of TRD patients may refuse or not tolerate conventional treatments, which may result in prolonged hospitalization.

Ketamine, a chemical derivative of phencyclidine, is an N-methyl-D-aspartate receptor antagonist (NMDA) and anesthetic agent that has been clinically available for more than 50 years [8]. Unlike traditional antidepressants, ketamine exerts its antidepressant effect through myriad pathways. Aside from blockade of interneuronal and excitotoxic extrasynaptic NMDA receptors, it also disinhibits pyramidal cells leading to a glutamate surge, activates prosynaptogenic α-amino-3-hydroxy-5-methyl-4- isoxazolepropionic acid (AMPA) receptors and synaptogenic intracellular signaling, increases γ-aminobutyric acid B (GABA-B) levels, and inhibits glycogen synthase kinase 3 (GSK-3B) [9,10,11,12,13,14]. Another characteristic that differentiates ketamine from traditional antidepressants is its rapid mood-enhancing effects, often appearing within four hours of administration. This is thought to be due to a glutamate surge that leads to a cascade of events involved in stress and mood modulation [8]. Ketamine is a versatile drug that has been historically used for analgesia and sedation, acute and chronic pain, and, more recently, for depression, acute suicidality, and post-traumatic stress disorder (PTSD) [8,15,16,17].

Ketamine has been shown to be effective in the management of TRD [18]. It has been studied as a pharmacological option in the treatment of both unipolar and bipolar depression in placebo-controlled trials and active-comparator studies, as well as in combination with conventional antidepressant agents [19,20]. Recently, a systematic review yielded a total of 22 randomized-controlled trials, 14 meta-analyses (1–16 studies per meta-analysis), and 2 Cochrane reviews on this topic [20]. A Cochrane review is a systematic review of research in health care and health policy that is published in the Cochrane Database of Systematic Reviews [21].

Despite this evidence, no currently published international guideline for the treatment of major depressive disorder recommends ketamine as treatment option for TRD. In 2017, Sancora et al. published a consensus paper on the use of ketamine in the treatment of mood disorders [22]. The authors provide information and suggestions to facilitate clinical decision making when using ketamine as an off-label treatment for psychiatric disorders, such as TRD [22]. The absence of clear recommendations is likely due to the lack of pivotal phase III clinical trials and knowledge gaps related to the long-term efficacy and safety of ketamine [22]. Economic factors make it unlikely that pivotal phase III clinical trials will ever occur [22].

There seems to be therapeutic inertia in adopting novel therapies in current practice for the treatment of TRD [23,24]. Limited treatment options and the challenges of offering novel evidence-based therapeutic alternatives to the most severely ill psychiatric patients could lead to delays in successful treatment and result in further suffering. Here, we highlight potential barriers to the accessibility of ketamine for TRD, and discuss the role of the pharmacist in facilitating ketamine treatment for patients with TRD.

## 2. Intravenous Ketamine

The administration of intravenous ketamine is an emerging practice in the management of TRD, and it involves the use of subanesthetic doses of ketamine. Unlike electroconvulsive therapy, which requires full general anesthesia, intravenous ketamine imparts less potential risk in terms of administration. A recent systematic review and meta-analysis included 22 randomized-controlled trials of intravenous ketamine of 0.5 mg/kg infused intravenously over 40–50 min for major depressive disorder, TRD, or bipolar depression [25]. The comparator arms were either placebo or active control with conventional antidepressant combinations. The randomized-controlled trials included patients who had inadequate response to at least one antidepressant trial of adequate doses and duration [25]. Response to treatment was defined as more than 50% reduction in symptom severity, measured by either the Hamilton Rating Scale for Depression, the Montgomery-Åsberg Depression Rating Scale, and/or the Quick Inventory of Depressive Symptomatology [25]. Clinical remission was defined as an absence of depressive symptoms, and was associated with depression scores less than or equal to nine on the Montgomery-Åsberg Depression Rating Scale or less than or equal to seven on the Hamilton Depression Rating Scale [25]. Overall, depression scores showed a statistically significant decrease following ketamine infusion relative to placebo [25]. Specifically, at 24 h following ketamine infusion, participants were seven times more likely to show a favorable clinical response and six and four times more likely to show clinical remission at 24 h and seven days post-infusion compared to control groups, respectively [25]. As is the case in electroconvulsive therapy, studies have shown that the effect size declines over subsequent weeks if no repeat intravenous ketamine treatments are administered, but it was observed that there was no sharp increase in suicidal ideation [16,25]. It is important to note that studies have not looked at the safety of long-term treatment with intravenous ketamine; the maximum duration in studies has been three months [20,25]. Common side effects of intravenous ketamine include dissociative symptoms, increase in heart rate, and a rise in blood pressure; these are all expected to normalize post-administration [16,25,26]. Occasional low blood pressure and premature ventricle beats may occur; however, termination of treatment has rarely been necessary [16,25,26].

Currently, very few hospitals in Canada provide intravenous ketamine treatment outside of experimental settings. In the Greater Toronto Area, Ontario, with a population of approximately 6.2 million, only one tertiary care hospital has started providing intravenous ketamine for TRD, which began in early 2021 [27]. In another highly populated metropolitan area in Canada, Vancouver, British Colombia, with an approximate population of 2.7 million, no hospital offers intravenous ketamine for TRD outside of experimental settings [28]. A handful of private clinics across Canada currently provide IV ketamine for TRD, with a charge of approximately CAD 750 per infusion [29]. The generally accepted protocol for the administration of IV ketamine for depression involves treatments administered two to three times a week for an estimated minimum of two weeks, then maintenance one to two times a month [22,26].

## 3. Intranasal Ketamine

### 3.1. Racemic Ketamine

Another route of administration for ketamine is intranasal. In recent years, the use of intranasal racemic ketamine has gained traction. The main advantages are higher bioavailability and faster onset than the oral route. It is also less invasive than intravenous administration [30]. Intranasal ketamine does not require continuous cardiac monitoring, and is therefore is less resource intensive than intravenous administration [15,31]. Although intranasal ketamine is more convenient, it has been associated with more variable absorption and can potentially result in higher peak serum levels and, theoretically, greater side effects [16,30].

In a meta-analysis comparing ketamine administered via different routes, McIntyre et al. concluded that the effect size was greatest for intravenous ketamine at two to six days compared to 24 h for intranasal racemic ketamine [16]. However, these results are difficult to interpret due to the heterogeneity present across studies. There has been one completed randomized-controlled trial, a small case series, and case reports looking at intranasal racemic ketamine. In the randomized-controlled trial, Lapidus et al. found that the estimated mean difference at 24 h post-treatment in the Montgomery-Åsberg Depression Rating Scale score between intranasal racemic ketamine and placebo was 7.6 ± 3.7 (N = 18) [15]. They also found that intranasal ketamine had a favorable tolerability profile, with minimal dissociative effects and comparatively small changes in hemodynamic parameters [15]. Currently, racemic ketamine is not commercially formulated for intranasal use (see Section 4).

### 3.2. Esketamine (Spravato^®^)

Esketamine is the active S(+) enantiomer of ketamine and is three to four times more potent than the R(−) enantiomer, which theoretically translates to lower doses required and fewer side effects in comparison to racemic ketamine [16,30,31,32]. Esketamine is commercially available, formulated as a single-use nasal spray device, marketed as Spravato^®^. Spravato^®^ is manufactured by Janssen Pharmaceuticals Inc., headquartered in Beerse, Belgium. The drug became available in Canada in July 2020 and in the United States (US) in March 2019 [33,34]. Esketamine is the S(+) enantiomer of ketamine. The commercially produced formulation and custom-designed intranasal delivery device eliminates concerns about product variability secondary to non-standardized compounding practices in producing racemic intranasal ketamine.

Esketamine’s efficacy and safety in TRD were assessed in five phase III studies: three four-week, placebo-controlled studies and two long-term trials [35]. Two four-week trials demonstrated no difference in the Montgomery-Åsberg Depression Rating Scale scores between placebo and esketamine in depressed patients [36,37]. The third trial, TRANSFORM-2, showed a difference of four points between esketamine and placebo at four weeks on the Montgomery-Åsberg Depression Rating Scale, favoring esketamine [38]. The long-term study, SUSTAIN-1, showed that esketamine is significantly beneficial in terms of extending time to relapse compared to placebo, with the risk of relapse decreasing in favor of intranasal esketamine by approximately 51% [39]. The other long-term study, SUSTAIN-2, had a primary outcome examining the treatment-emergent adverse events, most of which were mild to moderate, indicating esketamine as being well-tolerated [32,39].

## 4. Barriers to Treatment

The natural course of TRD is severe and often fatal [1]. Yet, there is still inertia in adopting emerging and novel therapies into practice. The uptake of evidence-based practice surrounding ketamine for TRD has been slow, and the Canadian healthcare system at large has been hesitant to translate the research evidence into practice. Given the limited treatment options for TRD, novel approaches should be considered and adopted into practice, and must be facilitated by a multi-disciplinary approach involving the physician or psychiatrist, the pharmacist, and the patient.

In the case of intravenous ketamine, very few hospitals across Canada currently provide this treatment, and only a handful of private clinics offer the treatment, albeit at a significant cost. Ketamine is a schedule I drug under the Controlled Drugs and Substances Act and a scheduled drug under the Narcotic Control regulations because it has significant potential for diversion. The use of ketamine for TRD is not currently approved by Health Canada or the Food and Drug Administration (FDA), and is therefore considered an off-label treatment [26]. The lack of Health Canada and FDA approval may lead to hospital and physician hesitancy to provide this treatment due to medical–legal liability associated with off-label treatments. In addition, most Canadian provincial government drug benefit programs do not cover intravenous ketamine treatment for the indication of TRD; thus, there is no billing code that exists for physician remuneration. Select third-party insurers, however, do provide coverage after special patient-specific consideration [32]. Intravenous ketamine must be administered in a controlled environment with basic cardiovascular (i.e., electrocardiogram, blood pressure) and respiratory (i.e., oxygen saturation or end-tidal CO_2_) function [22]. There should be ongoing assessments of patients’ physiological and mental status during the infusion (e.g., blood pressure and heart rate should be reported every 15 min) [22]. The facility should also have means of delivering oxygen to patients with reduced respiratory function and rapidly manage sustained alterations in cardiovascular function [22]. It is also recommended that a licensed clinician, who can administer a controlled substance and who has Advanced Cardiac Life Support certification, provide the treatment [22]. There may also be local requirements regarding the initiation and administration of the infusion, depending on the policies of specific institutions and professional colleges. For example, many academic hospitals in Toronto, Canada, also mandate that an anesthesiologist or an anesthesia assistant administer the infusion. These requirements, while necessary, create logistical and financial challenges for publicly funded institutions, further limiting the accessibility of ketamine for patients with TRD.

As mentioned above, intranasal ketamine is not commercially formulated for intranasal use. Specialized compounding pharmacies are needed to produce the intranasal product from powder or parenteral solution, dispensed with a nasal drug delivery device for administration. This, however, introduces potential variability in the final formulation, since there is no mandatory standardization in compounding methods, formula, or in the intranasal delivery devices used for administration. Health Canada states that the compounding of drugs should be reserved for situations where there is a need for therapy that is lacking in commercial product availability and should not act as a replacement for an approved drug product [40]. Since intranasal eskatamine, Spravato^®^, is now available, prescribers now have two intranasal treatment options, but may not understand the clinical or logistical implications of each.

Intranasal eskatamine, Spravato^®^, is the only ketamine product to have received Health Canada and FDA approval for the treatment of TRD. This eliminates the liability risks which are associated with prescribing other ketamine products for TRD. However, it is not currently covered by most Canadian provincial government drug benefit programs or hospital formularies. The cost is approximately CAD 750 per 56 mg dose and CAD 1128 per 84 mg dose [32]. The dosing regimen is 56 mg or 84 mg twice weekly for four weeks, followed by once weekly for four weeks, then once weekly or once every other week for ongoing maintenance therapy [34]. This means an approximate minimum annual cost of CAD 24,000 for the lower dose regimen and CAD 36,100 for the higher dose regimen. For comparison, the median after-tax income of Canadian families and unattached individuals was approximately CAD 61,400 in 2018 [41]. The cost of treatment significantly limits accessibility to this drug and the ability to maintain long-term treatment as an outpatient for many Canadians. Low socioeconomic status has been found to be significantly associated with higher prevalence of depression. Thus, inaccessibility due to cost disproportionately impacts the population that may need this treatment the most [42].

Aside from cost, there are specific logistics associated with prescribing and dispensing Spravato^®^ that also limit patient access. In Canada, Spravato^®^ can only be prescribed by psychiatrists and dispensed by pharmacists who are certified by, and enrolled in, a Risk Evaluation and Mitigation Strategy program established by Janssen Pharmaceutical Inc. headquartered in Beerse, Belgium, also known as the Janssen Journey™ Program [33]. The Janssen Journey™ program is a controlled distribution program aimed at mitigating the risks of adverse outcomes (i.e., sedation, dissociation, and hypotension) and the risk of misuse and abuse [33]. Spravato^®^ can only be dispensed to sites of care where patients self-administer the product under the direct supervision of a health-care professional and are monitored by a healthcare professional post-administration [33]. The program allows psychiatrists to prescribe Spravato^®^ in a hospital or community setting as long as the other administration requirements are met [33]. This unintentionally creates difficulties in continuing therapy initiated in a secondary or tertiary care center, since the receiving community psychiatrist and pharmacist must also be enrolled in this program. This is a barrier to continuity of care and adds another layer of complexity to ensuring patients receive the care they need.

## 5. Pharmacist Role

Pharmacists have a critical role in ensuring access to quality care. This includes dissemination of evidence supporting pharmacological treatments and facilitating translation to current practice. Pharmacists regularly collaborate with prescribers to optimize drug therapy, address modifiable barriers to treatment, and triage access to medications during transitions of care.

With respect to the use of intravenous ketamine for TRD, the role of the pharmacist in interpreting and disseminating evidence, as well as highlighting drug legislation, could help prescribers understand why certain barriers exist and how they could be overcome. The administration of intravenous ketamine is invasive and requires appropriately skilled medical staff to administer and monitor the patient [22]. In Canada, this treatment is currently only administered in an inpatient setting or in a specialized ketamine infusion clinic. Pharmacists can help to ensure seamless transitions of care between inpatient and outpatient treatment, and thereby contribute to reducing the probability of readmissions [43,44]. Furthermore, pharmacists will assess for drug–drug interactions, drug-disease interactions (i.e., patients with substance use disorder or history of psychosis), and facilitate safe preparation of the medication [22].

In the case of intranasal ketamine, prescribers now have options, but may not appreciate the clinical and practical differences between prescribing compounded racemic ketamine and esketamine (Spravato^®^). Although identical in route of administration, they differ in terms of chemistry, efficacy, safety, acquisition, and cost. Pharmacists can offer their unique expertise in these areas, highlighting a new opportunity for increased pharmacist involvement in the care of this patient population.

For esketamine (Spravato^®^) therapy, one of the barriers to treatment is the manufacturer’s restrictions in terms of prescribing and dispensing the product. In Canada, intranasal esketamine is only accessible via a controlled distribution program called the Janssen Journey™ program [33]. Before the patient can begin therapy, three parties (the patient, the prescriber, and the pharmacy) must be registered with and receive online training via this program [33]. This restriction serves as a safeguard to prevent misuse and diversion. However, unlike drug costs and drug benefits, this barrier to access could be mediated and efficiently managed by a pharmacist in order to maximize patient access. Logistics in drug prescribing and dispensing should not prevent a patient from receiving a potentially life-saving treatment. For Spravato^®^, the specific requirements for dispensing may not be widely appreciated. This could result in delayed initiation of treatment, or continuing treatment if patients are admitted or discharged from hospital. It could also result in unintentional gaps in continuing treatment if a patient is admitted to, or discharged from, hospital without having a prearranged pharmacy and pharmacist registered with the program to continue dispensing the treatment. Pharmacists could take a lead in engineering and implementing systems and strategies to coordinate and streamline Spravato^®^ prescribing and dispensing. This will involve providing educational outreach and strengthening information systems between hospital pharmacists and community pharmacists regarding the requirements associated with prescribing and dispensing Spravato^®^ therapy. Such a network could facilitate and reinforce knowledge sharing, discussion, and aid in navigating and resolving potential logistical challenges related to the drug utilization process for Spravato^®^.

A pharmacist is an integral part of a treatment team, and an invaluable asset in facilitating logistics and providing drug information to both patients and physicians [45]. As pharmacists’ scope-of-practice expands, current and ongoing research has illustrated their roles in leading initiatives and establishing programs that aim to bridge the pharmacotherapy gaps in healthcare services [46,47,48]. To overcome barriers to providing novel and effective treatments in treatment-resistant depression, effective pharmacy-led leadership and governance is imperative. The limitations and barriers to ketamine and esketamine treatment become evident when translating research into practice. Extending the reach of these novel psychiatric treatments in both tertiary and primary care settings creates an emerging role for pharmacists in the collaborative effort to better manage treatment-resistant depression.
